# Detection of Muscle Activation during Resistance Training Using Infrared Thermal Imaging

**DOI:** 10.3390/s21134505

**Published:** 2021-06-30

**Authors:** Haemin Jung, Jeongwung Seo, Kangwon Seo, Dohwi Kim, Suhyun Park

**Affiliations:** 1 Department of Electronic and Electrical Engineering, Ewha Womans University, Seoul 03760, Korea; goalssla12@cau.ac.kr; 2 School of Electrical and Electronics Engineering, Chung-Ang University, Seoul 06974, Korea; cyh4681@cau.ac.kr (J.S.); skangwon@naver.com (K.S.); 3 Thermoeye, Seoul 06979, Korea; oranss@thermoeye.co.kr

**Keywords:** infrared thermal imaging, muscle activation, skin temperature, deep learning, brachium

## Abstract

Infrared thermal imaging has been widely used to show the correlation between thermal characteristics of the body and muscle activation. This study aims to investigate a method using thermal imaging to visualize and differentiate target muscles during resistance training. Thermal images were acquired to monitor three target muscles (i.e., biceps brachii, triceps brachii, and deltoid muscle) in the brachium while varying the training weight, duration, and order of training. The acquired thermal images were segmented and converted to heat maps. By generating difference heat maps from pairs of heat maps during training, the target muscles were clearly visualized, with an average temperature difference of 0.86 °C. It was observed that training order had no significant effect on skin surface temperature. The difference heat maps were also used to train a convolutional neural network (CNN) to show the feasibility of target muscle classification, with an accuracy of 92.3%. This study demonstrated that infrared thermal imaging could be effectively utilized to locate and differentiate target muscle activation during resistance training.

## 1. Introduction

Resistance training exercises cause a subject’s muscles to contract against an external resistance, which eventually strengthens and builds muscle endurance. Manipulating training variables (e.g., load weight, recovery period, and lifting speed) can produce a wide range of training stimuli [1]. Thus, various strategies have been explored to quantify the effects of resistance training. Marston et al. proposed a metric called exercise density and investigated its relation to a physiological marker of internal training intensity along with traditional measures (e.g., number of repetitions and volume load) of external training intensity [2]. Another study calculated mechanical work to quantify external resistance training volume during a resistance training session [3]; however, this process is time consuming and requires specialized equipment, thereby limiting its practical application [4]. Although quantitative amounts of training can be measured, the direct effects of training on the muscles are not considered. Surface electromyography (sEMG) has been widely used to quantify muscle effort during resistance exercises via direct monitoring of muscle activation [5]. By evaluating the zero-crossing levels of sEMG signals, assessments of a subject’s fatigue endurance have been presented [6]. However, sEMG requires placement of surface electrodes on the target muscles, which impede the subject’s movements during training. In addition, there are various factors that affect impedance measurements between muscles and sEMG electrodes, such as biological tissue composition, distance between muscle and electrodes, and size of the electrodes [7,8].

During resistance training, heat is dissipated from muscle contraction owing to metabolism and blood perfusion. Therefore, some studies have investigated the relationship between temperature changes and training [9,10,11,12,13,14]. A previous study showed that prolonged exercise increased the amount of heat dissipated from muscle [9]. Several works have utilized thermistors to investigate changes in the local temperature of muscles after exercise [10]; however, these measurements were invasive, as the thermistors were inserted into the subjects’ veins. Owing to its noninvasive and noncontact nature, infrared thermal imaging has been widely used for various biomechanical applications [11,12,13]. Using infrared thermal imaging, several studies have investigated skin surface temperature changes related to muscle activation [9,15,16,17,18,19]. It is known that human skeletal muscles have a mechanical efficiency of 30–65%, and there is heat dissipation (i.e., temperature change) related to muscle contraction [9]. Zagrodny et al. showed that there is a correlation between sEMG and thermal characteristics of the skin surface [15]. Daud et al. also showed a correlation between sEMG signals and skin temperature measured from thermal imaging [16]. In [17], the authors observed that the temperature of the quadriceps before and after training showed substantial agreement with changes in skin temperature following physical exercise. Another study investigated localized heating of limbs in individuals of different age groups [18]. Furthermore, temperature changes from high-intensity exercise associated with muscle fatigue have also been investigated [18,19]. Therefore, previous studies have proven that the change of skin surface temperature measured from thermal imaging is highly correlated with the isolation of muscle activation. Although the correlation between muscle activation and thermal characteristics has been demonstrated in some of the previous studies, these were limited to a single region or muscle. Thus, it was necessary to investigate a method to locate and differentiate muscle activation during training.

This study aimed to investigate the use of infrared thermal imaging to assess the isolation of muscle activation during resistance training. Based on previous studies, it was assumed that the skin surface temperature measured from thermal imaging can be directly related to muscle activation. Thermal images were acquired during resistance training while varying the training conditions. The thermal images were then processed and analyzed to visualize muscle activation. To evaluate the performance of the proposed method, a deep-learning model was trained and tested on the processed thermal images for classification of the target muscle activation. The advantage of this study is that the activations of different muscles can be located and differentiated by utilizing the thermal images acquired in a noninvasive and noncontact manner.

## 2. Materials and Methods

### 2.1. Experimental Setup

**Figure 1:**
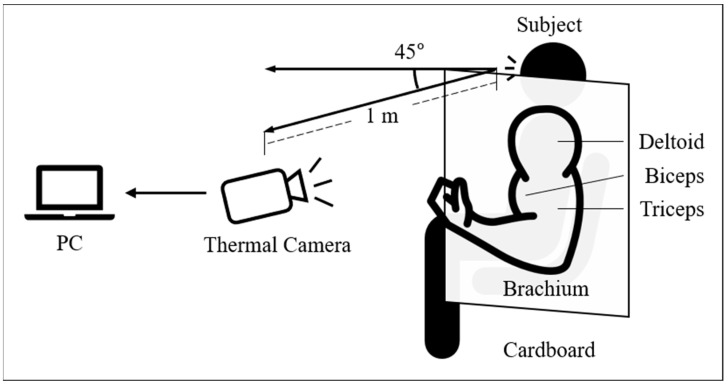
Experimental setup for detection of muscle activation. Thermal images of the brachium were obtained using a thermal camera during the training sessions.

Figure 1 shows the experimental setup used to detect muscle activation. Thermal images were acquired using a thermal camera (A65SC, FLIR systems Inc., Wilsonville, OR, USA) with a resolution of 640 × 512 pixels and a thermal sensitivity of <0.05 °C. The acquired thermal images were converted to temperature values using ResearchIR software (FLIR systems Inc., Wilsonville, OR, USA). Cardboard with a square-shaped hole was placed between the arm and body of the subject. The distance between the camera and subject was set to 1 m. The camera was placed at a 45° angle from the frontal body to ensure that the target arm muscles were within the camera imaging frame.

The three major muscles in the brachium (i.e., upper arm), which are biceps brachii (hereinafter called biceps), triceps brachii (hereinafter called triceps), and deltoid muscle (hereinafter called deltoid), were employed as the target muscles. To stimulate these target muscles, arm curl, kick back, and single-arm lateral raise exercises were performed for the biceps, triceps, and deltoid, respectively. The experiments were conducted on five male participants, who were selected among volunteers based on their ability to endure the training strength required in this study, at ambient temperature. The average age, height, and weight of the subjects were 26.2 ± 1.32 years, 175 ± 0.89 cm, and 67.4 ± 2.79 kg, respectively. The subjects exposed their brachium to air for 15 min before thermal image acquisition.

### 2.2. Experimental Procedures

The subjects performed training movements to activate each of the target muscles. All training was performed at the rate of 15 repetitions per minute and timed with a metronome to ensure periodicity of movements. The total number of sets per session, recovery time, and repetitions per set were varied between experiments. Three different experiments were conducted to investigate the effects of isolated resistance training on the skin-surface temperature. A summary of the three training conditions assessed in this study is shown in Table 1.

To investigate the effects of weight variation (experiment I) on the subject’s skin surface temperature changes, the biceps was chosen as the target muscle. A single subject performed one set of full arm-curl sessions, where each set included 15 repetitions (i.e., 60 s duration) to ensure that the training session fully stimulated the target muscle. This was followed by 5 min of recovery. Thermal videos of two sessions were acquired, while the training sessions were performed using 5 kg and 10 kg dumbbells separately. In addition, the effects of consecutive sets of training (experiment II) on the subject’s skin-surface temperature were investigated for the biceps. The subject performed three sets of eight repetitions (i.e., 36 s duration including passive (no movement with weight) training for 4 s). Each set was followed by 92 s of recovery. To fully stimulate the target muscle, a 10 kg dumbbell was used. Thermal video of the entire session was recorded. Lastly, muscle activation from different target muscles (experiment III) was investigated. To observe the effects of the order of training on different target muscles, three sessions with three different training orders, corresponding to activation of the three different target muscles, were employed. The selected training orders were order #1: arm curl–lateral raise–kickback; order #2: lateral raise–kickback–arm curl; and order #3: kickback–arm curl–lateral raise; hence, the corresponding muscle activations were biceps–deltoid–triceps; deltoid–triceps–biceps; and triceps–biceps–deltoid. Each subject performed three sets of 12 repetitions (i.e., 48 s duration), followed by 92 s of recovery for each session. Each subject chose either the 5 kg or 10 kg dumbbell according to their ability to withstand the full training session. Each training session was repeated for both the left and right arms, and a total 70 sessions were selected.

### 2.3. Data Processing

**Figure 2:**
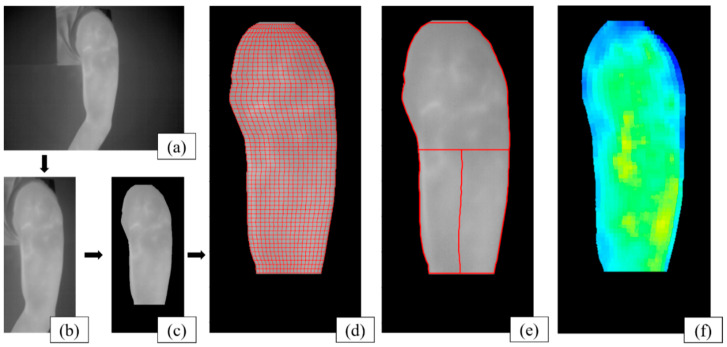
Visualization of the process of analysis of a single image: (**a**) original thermal image, (**b**) cropped image around the brachium, (**c**) image after segmentation, (**d**) 20 × 60 divided regions, (**e**) heat map with three selected regions for the target muscles, and (**f**) colorized heat map of the target muscles.

All images were aligned so that the biceps were located on the left sides of the images (Figure 2a). Then, the brachium area was manually cropped (Figure 2b). After applying a moving average filter, Otsu’s method [20] for global thresholding was applied to segment the brachium areas (Figure 2c). Further segmentation was manually performed, if necessary. The region of interest (ROI) was then evenly divided into 20 horizontal and 60 vertical sections (Figure 2d). A heat map was generated by calculating the mean temperature from each divided region in the ROI (Figure 2e) and displayed using a color map (Figure 2f).

To detect muscle activation, the thermal images at the beginning and end of each set were chosen from each training set. Thus, a total of six images were used from the three sets in a single session. Using the thermal images, heat maps were generated and denoted as (S1), (S2), and (S3) and (E1), (E2), and (E3) for the beginning and end of each set, respectively. Thus, a total of 420 heat maps were generated out of the 70 sessions.

To analyze the temperature changes from the target muscle regions, each heat map was divided into three regions, as shown in Figure 2e. Based on the positions of the muscles, it was assumed that the upper-half region was the deltoid, the lower-left quadrant was the biceps, and the lower-right quadrant was the triceps. The mean value of each region represents the temperature of the corresponding muscle. Statistical analyses were performed using the Kruskal–Wallis test to examine the temperature difference for each target muscle between the three training orders.

### 2.4. Classification of Target Muscle Activation

The ResNet-18 [21] network was used to classify target muscle activation using the difference heat maps (i.e., temperature differences) from the proposed method. To train the network, a total of 134 difference heat maps were generated from the differences between the heat map pairs, which are (S1-E3) and (S1-S3). The target classification was divided into four classes, namely biceps, triceps, deltoid, and vague. The “vague” class implies that no valid muscle activation was observed. The test set was 10% of randomly selected data, thus resulting in 121 training data and 13 test data. The summary of the dataset is shown in Table 2. For augmentation of the training data, the heat maps were processed by rotation from −1° to 1° in steps of 1°, Gaussian noise addition with a mean of 0.07, scaling by 0.9 and 1.1 times, and resizing to a resolution of 244 × 244 pixels, resulting in 2178 training images. The network model was initialized using the pretrained weights with the ImageNet dataset [22] for ResNet-18 provided in MATLAB (Mathworks Inc., Natick, MA, USA). Then, the model was trained by finetuning with a learning rate of 1 × 10^−4^ and batch size of 16 for 50 epochs. The Adam optimizer and cross-entropy loss were used for training.

## 3. Results

### 3.1. Effects of Weight, Duration, and Muscle Activation for Training

**Figure 3:**
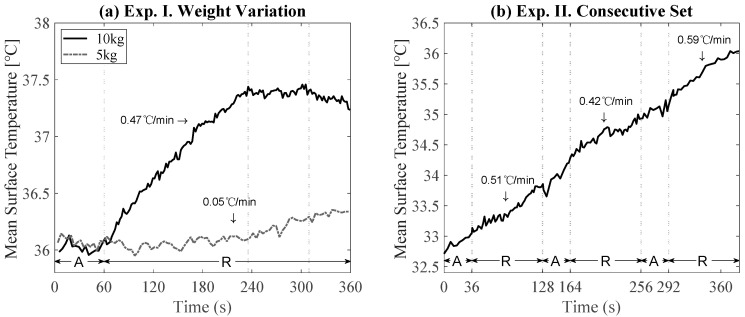
Change in skin surface temperature from biceps in (**a**) a session of a single set with 5 kg and 10 kg dumbbells and in (**b**) a session of three consecutive sets with a 10 kg dumbbell. (“A” and “R” denote active training and recovery periods, respectively.).

Figure 3 shows the changes in skin surface temperatures from the biceps with varying weights (experiment I (Figure 3a) and in consecutive sets of training (experiment II (Figure 3b). For the measurement with a 5 kg dumbbell (dotted line in Figure 3a), the initial biceps skin temperature was measured as 36.07 °C. After one minute of training, the biceps skin temperature was still 36.07 °C. After five minutes of recovery, the skin temperature reached a maximum of 36.34 °C. While the average rate of increase of the skin surface temperature on the biceps during training was negligible, the skin surface temperature during recovery was 0.05 °C/min. With a 10 kg dumbbell (solid line in Figure 3a), the skin surface temperature was 35.98 °C initially and 36.11 °C at the end of training. After five minutes of recovery, the temperature reached up to 37.24 °C. The average elevation rate was 0.13 °C/min during exercise and 0.47 °C/min during 3 min of recovery. It was therefore observed that the increase in the skin surface temperature occurred during recovery. Moreover, the skin temperature reached a plateau and decreased after enough recovery time (3 min in this study). The difference in the average elevation rate during 3 min of recovery with the 5 kg and 10 kg weights was 0.46 °C/min. Figure 3b shows skin surface temperature changes during three consecutive sets of training with recovery times between each set. The temperature gradually rose during rest, which matches with the results from a single set of training in Figure 3a. Further, it was observed that the skin surface heated up as more sets of training were performed. The overall average elevation rate was 0.52 °C/min. In each recovery period, the average elevation rates of the skin surface temperatures were 0.51, 0.42, and 0.59 °C/min, as shown in Figure 3b. The mean skin surface temperatures during exercise were 32.90, 34.00, and 35.07 °C for each training set. The mean skin surface temperatures during recovery were 33.42, 34.65, and 35.64 °C after each training set.

**Figure 4:**
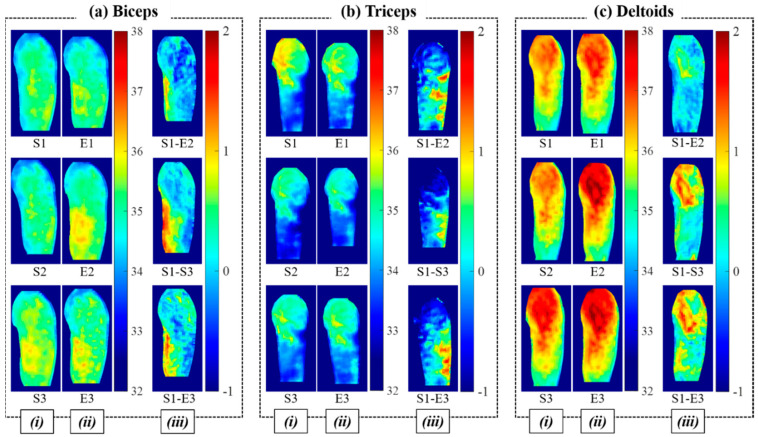
Heat maps from muscle activation of (**a**) biceps, (**b**) triceps, and (**c**) deltoid. Column (*i*): heat maps of the beginning of each training set (S1-S3), column (*ii*): heat maps of the end of each training set (E1-E3) from a single session, and column (*iii*): difference heat maps from (S1-E3), (S1-S2), and (S1-S3) pairs.

Figure 4 shows examples of the heat maps for the three different muscle activations (i.e., (a) biceps, (b) triceps, and (c) deltoid) from experiment III. Column (*i*) shows the heat maps of the beginning of each training set (S1-S3), column (*ii*) shows the heat maps of the end of each training set (E1-E3) from a single session, and column (*iii*) shows the difference heat maps from the (S1-E2), (S1-S3), and (S1-E3) pairs. The difference heat maps are shown in Figure 4a–c (*iii*).

Difference heat maps were generated from various heat map pairs, including (S1-E3), (S1-S3), (S1-E2), (S1-S2), and (S1-E1). Table 3 shows the numbers of pairs that produce the highest temperature differences among the various heat map pairs from each session. The average difference for S1-E3 of the entire collected data was 0.86 °C, and the highest temperature difference was 0.97 °C. The higher the temperature difference, the clearer the differentiation of the selected muscles. It was observed that 85% of the highest temperature difference was acquired from the heat map pairs of the first and third sets of each session.

**Figure 5:**
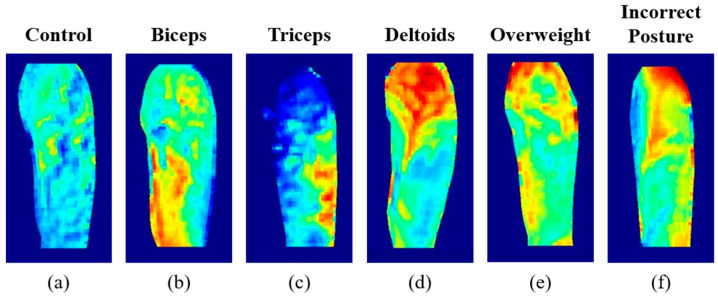
Heat map examples from (**a**) control from passive training, (**b**–**d**) proper training for biceps, triceps, and deltoids, respectively, (**e**) over-weighted training, and (**f**) incorrect posture of training.

Figure 5 shows various heat map examples generated from the experiments in this study. Figure 5a is the control case where the subjects performed training with no weights (i.e., passive training); no local heating was observed in this case. Figure 5b–d clearly demonstrates local heating of the target muscles with proper training for each target muscle activation. Figure 5f shows an example of unexpected muscle activation from over-weighted training, where the subject swung the elbow excessively while targeting the biceps. Additionally, in the case of Figure 5e, the subject performed an inaccurate kick-back session. The resulting images show that the temperature increased throughout the brachium, especially at the posterior deltoid (i.e., upper-right area).

### 3.2. Statistical Analysis

**Figure 6:**
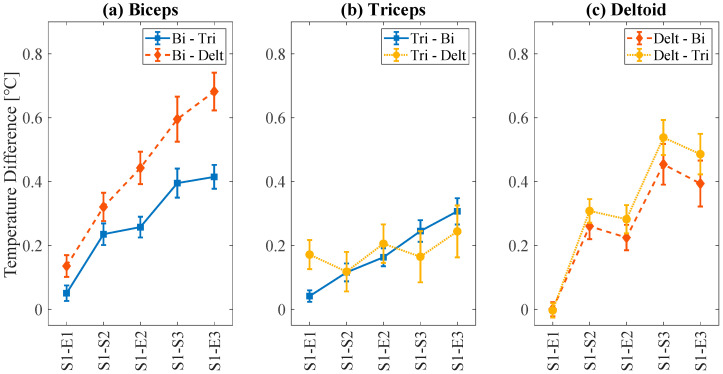
The average temperature difference between target muscles and non-target muscles when targeting (**a**) biceps, (**b**) triceps, and (**c**) deltoid. Error bars denote standard deviation.

Figure 6 shows the overall average of temperature differences between the regions of target and nontarget muscles during the exercise session. Overall, the heat map pair (S1)-(E3) showed the most significant temperature difference, as analyzed from Table 2. The results in Figure 6 match well with those in Figure 3, where the surface temperature of the target muscle gradually increased during a session with three training and rest periods. In the case of the biceps (Figure 6a), the temperature difference was higher when compared with the deltoid (dotted line) than with the triceps (solid line). For the triceps case (Figure 6b), the overall temperature difference was lower compared to the other cases. For the deltoid case (Figure 6c), the trend with the triceps (solid line) was similar to that of the biceps (dotted line).

**Figure 7:**
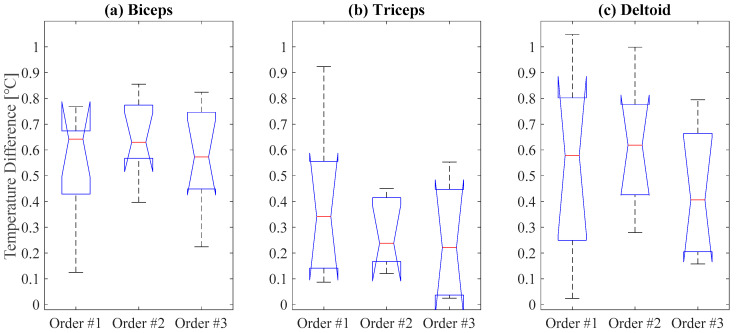
Temperature differences for target muscles (**a**) biceps, (**b**) triceps, and (**c**) deltoid from three different training orders (order #1: biceps–deltoid–triceps, order #2: deltoid–triceps–biceps, and order #3: triceps–biceps–deltoid).

Figure 7 shows the median and variance values of the temperature differences for each target muscle from the three different training orders. The Kruskal–Wallis test showed that no significant differences (*p*-value = 0.57, 0.62, and 0.40 for biceps, triceps, and deltoid, respectively) were found among the three different training orders. The results demonstrate that the order of muscle activation had insignificant effects on the temperature changes during exercise.

### 3.3. Classification of Muscle Activation

**Figure 8:**
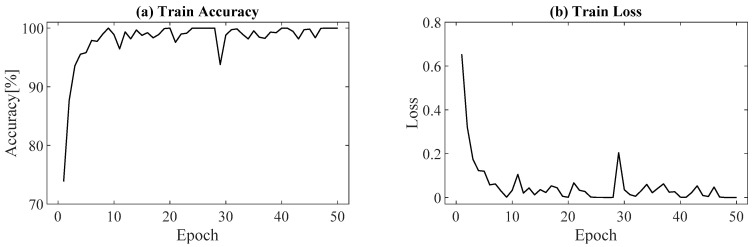
Summary of network training: (**a**) training accuracy and (**b**) loss function.

**Figure 9:**
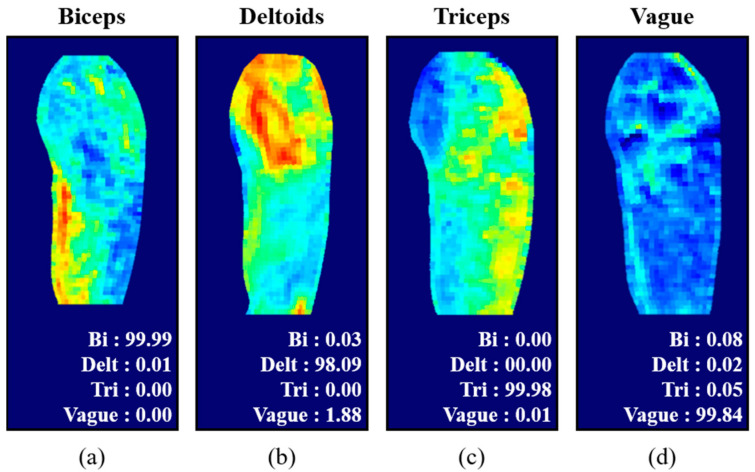
Classification of muscle activation. (**a**–**d**): test heat maps of three different muscle activations and vague case with predicted probabilities from the network, respectively.

The training accuracy and loss function of the network training are shown in Figure 8. After 50 epochs, 100% training accuracy and 92.3% test accuracy were achieved. Figure 9 shows the prediction probabilities for the classification of difference heat maps for various muscle activations. Each target muscle activation showed the highest prediction probability from the correct class (biceps: 99.99% (Figure 9a), deltoid: 98.09% (Figure 9b), triceps: 99.98% (Figure 9c), vague: 99.84% (Figure 9d). Although insufficient training and test datasets were utilized, the results clearly demonstrate that classification of muscle activation can be easily achieved from the trained network using the difference heat maps.

## 4. Discussion

This study demonstrated the correlation between isolated resistance training of three separate muscles of the brachium and temperatures of their corresponding locations on the skin surface. Three muscles of the brachium (i.e., biceps, triceps, and deltoid) were selected owing to their easy isolation for resistance training movements and the anatomy of the muscles distinguishable under thermal imaging. The main findings of this study are as follows: (1) weight variation during training affects the change in skin surface temperature; (2) consecutive training sets increase heating of the skin surface; and (3) the order of training has an insignificant effect on isolating muscle activation.

It was observed that temperature differences in the thermal images were highest mostly between the start and end of training sessions (Table 2). This does not match well with results from previous studies, which considered training with high intensity and duration leading to perspiration and muscle fatigue [23,24,25]. In this study, the effects from perspiration and muscle fatigue were minimized using recovery time between the training sets. Overall, there were relatively subtle temperature changes during training compared to that during recovery time, as shown in Figure 3 and Figure 4. This can be explained by the process of human metabolism. When muscles produce energy by movement, PCr hydrolysis is initially dominant (~10 s), followed by glycolysis (~50 s). Subsequently, oxidation will dominate energy production [9]. As heat production occurs mainly during the oxidation process, exercise durations longer than 60 s are expected to contribute to heat production. In this study, the training time for each set was up to 60 s, so the oxidation process was not considered. As it is known that only 2% of heat produced from the muscle is instantly dissipated through the skin while the rest is accumulated in the muscle and slowly perfused through skin, inducing vasodilation near the activated muscle [10], this heat transfer causes delays in temperature change on the skin surface. Thus, it is expected that the temperature rise during the recovery time would be more noticeable than that during training, as observed in this study.

Overall, the results in Figure 6 show that the biceps region exhibited the most significant rise in temperature. This can be explained by the presence of major veins (i.e., cephalic vein) in the biceps region. The heat accumulated during training was dissipated through the veins. In contrast, in other regions with no major veins, heat dissipation was relatively low (Figure 6b,c). Owing to the thickest skinfold with no major veins, the heat dissipation from the triceps region was the lowest among the three muscles [26], which was also observed in this study (Figure 6b). This is presumably because the deltoid muscles function as synergist muscles during motion. Although the overall temperature increased, there were several temperature decreases for the deltoid (Figure 6c). During the lateral raise motion to activate the deltoid muscle, the subjects swung their arms away from the body until their arms became parallel with the floor. This movement may cause cooling via respiration.

As shown in Figure 5a–f, accurate training postures result in localized heating of the skin directly above the target muscles. Thus, accurate training postures for isolated resistance training can be clearly visualized using thermal imaging. Utilizing the difference heat maps generated in this study, a convolutional neural network (ResNet-18 in this study) was trained and tested for classification of muscle activation. Although there were not sufficient data to discuss the accuracy or performance of the network, the test results of the network clearly demonstrate the feasibility of the proposed approach to differentiate muscle activation. The proposed technique can be further utilized in physical therapy, which should be performed with proper muscle activation.

In this study, the number of subjects was limited, and the number of variations was small. There are various conditions of the subject, such as body mass composition, muscle mass, training skill, color and thickness of skin, thickness of skinfold, and age, that may affect the result of this study. Thus, further investigations by performing experiments on various subject groups are necessary [24,27]. In order to achieve repeatability of the measurements, it is necessary to minimize changes in distance and angle between the camera and the subject. Thus, motion tracking of the target area could be implemented. Although the proposed approach is a noninvasive and noncontact method, the surface of the skin needs to be exposed during the measurement. As only two absolute loads (5 and 10 kg) were utilized, which can induce different relative intensities of work depending on the subject’s muscle strength, it is also necessary to consider the absolute intensity of work correlated to muscle activation. Further investigation on validating the accuracy and efficacy of the isolated muscle training sessions with the proposed approach will be our future work.

## 5. Conclusions

The activation of three different brachium muscles during resistance training was detected using infrared thermal imaging and analyzed using heat maps. The proposed approach in this study demonstrated that the differences in heat maps could be used to effectively differentiate the target muscles. Further investigations are necessary to validate this approach on subject groups with various conditions and to expand it to other target muscles. Future studies will also seek to improve the accuracy and efficacy of the proposed approach for muscle isolation.

## Figures and Tables

**Table 1:** Summary of training conditions for the experiments (#: Number).

Experiment	Training Weight [kg]	Recovery Time [s]	# of Sets per Session	# of Sessions	# of Repetitions per Set	# of Subjects	Target Muscle
I. Weight	5 and 10	300	1 set	1	15	1	Biceps
II. Duration	10	92	3 sets	1	8	1	Biceps
III. Muscle	5 or 10	92	3 sets	3	12	5	All Three

**Table 2:** Summary of the dataset used for training and test for classification.

Dataset	Biceps	Triceps	Deltoid	Vague	Total
Train	33	19	31	38	121
Test	4	3	2	4	13

**Table 3:** Counts of different periods showing the highest temperature differences from heat map pairs.

Heat Map Pairs	Count	Percentage
(S1-E3)	36	51%
(S1-S3)	24	34%
(S1-E2)	4	5%
(S1-S2)	2	3%
(S1-E1)	4	5%

## Data Availability

The data presented in this study are available upon request from the corresponding author.
